# Interface Reversible Electric Field Regulated by Amphoteric Charged Protein-Based Coating Toward High-Rate and Robust Zn Anode

**DOI:** 10.1007/s40820-022-00969-4

**Published:** 2022-11-10

**Authors:** Meihua Zhu, Qing Ran, Houhou Huang, Yunfei Xie, Mengxiao Zhong, Geyu Lu, Fu-Quan Bai, Xing-You Lang, Xiaoteng Jia, Danming Chao

**Affiliations:** 1 College of Chemistry, Jilin University, Changchun, 130012 People’s Republic of China; 2 State Key Laboratory of Integrated Optoelectronics, College of Electronic Science and Engineering, Jilin University, Changchun, 130012 People’s Republic of China; 3 Key Laboratory of Automobile Materials, Ministry of Education, School of Materials Science and Engineering, Jilin University, Changchun, 130022 People’s Republic of China

**Keywords:** Silk fibroin coating, Zn anode, Amphoteric charge, Interfacial engineering, Aqueous zinc-ion batteries

## Abstract

Metallic interface engineering is a promising strategy to stabilize Zn anode via promoting Zn^2+^ uniform deposition. However, strong interactions between the coating and Zn^2+^ and sluggish transport of Zn^2+^ lead to high anodic polarization. Here, we present a bio-inspired silk fibroin (SF) coating with amphoteric charges to construct an interface reversible electric field, which manipulates the transfer kinetics of Zn^2+^ and reduces anodic polarization. The alternating positively and negatively charged surface as a build-in driving force can expedite and homogenize Zn^2+^ flux via the interplay between the charged coating and adsorbed ions, endowing the Zn-SF anode with low polarization voltage and stable plating/stripping. Experimental analyses with theoretical calculations suggest that SF can facilitate the desolvation of [Zn(H_2_O)_6_]^2+^ and provide nucleation sites for uniform deposition. Consequently, the Zn-SF anode delivers a high-rate performance with low voltage polarization (83 mV at 20 mA cm^−2^) and excellent stability (1500 h at 1 mA cm^−2^; 500 h at 10 mA cm^−2^), realizing exceptional cumulative capacity of 2.5 Ah cm^−2^. The full cell coupled with Zn_x_V_2_O_5_·nH_2_O (ZnVO) cathode achieves specific energy of ~ 270.5/150.6 Wh kg^−1^ (at 0.5/10 A g^−1^) with ~ 99.8% Coulombic efficiency and retains ~ 80.3% (at 5.0 A g^−1^) after 3000 cycles.

**Supplementary Information:**

The online version contains supplementary material available at 10.1007/s40820-022-00969-4.

## Highlights

- Alternating positively and negatively charged surface controlled by pH expedites and homogenizes Zn^2+^ flux, endowing the Zn- silk fibroin (SF) anode with low polarization voltage and stable stripping/plating.
- Experimental analyses with theoretical calculations suggest that SF coating facilitates the desolvation of [Zn(H_2_O)_6_]^2+^ and provides nucleation sites for uniform deposition.
- Symmetric battery of Zn–SF anodes delivers high-rate performance (up to 20 mA cm^−2^) and excellent stability (1500 h at 1 mA cm^−2^; 500 h at 10 mA cm^−2^) with cumulative capacity of 2.5 Ah cm^−2^.

**Supplementary Information:**

The online version contains supplementary material available at 10.1007/s40820-022-00969-4.

## Graphical abstract

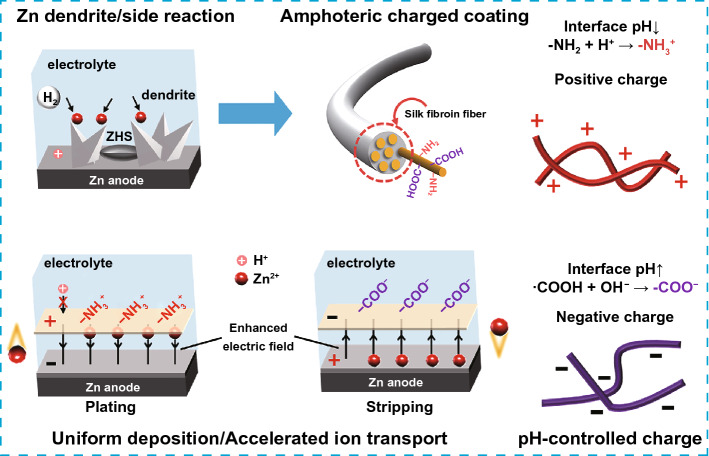

## Introduction

Aqueous zinc-ion batteries (AZIBs) hold significant potential for large-scale energy storage systems, benefiting from good safety, low cost, and environmental compatibility [1–3]. Although AZIBs have witnessed rapid development in cathode design, poor cycling stability of Zn anode is still a severe problem caused by dendrite growth, hydrogen evolution reaction, and corrosion by-products (Zn_4_SO_4_(OH)_6_·xH_2_O, ZHS) [4, 5]. Hence, many approaches have been proposed to stabilize Zn anodes, including anode construction (3D current collector/alloy) [6, 7], electrolyte optimization (additives/highly concentrated salts) [8–10], and interfacial protective coatings [11–14]. The anode construction method significantly decreases the local current density while increasing the specific surface area [15, 16]. Moreover, the introduction of zincophilic sites is promising to reduce the nucleation energy barrier and homogenize Zn^2+^ flux. The electrolyte optimization technique adjusts the Zn^2+^ solvation structure and weakens water activity, facilitating the Zn plating/stripping kinetics and the uniform distribution of Zn^2+^ [9].

As an alternative strategy, coating the Zn anode surface with an interfacial protective layer can restrict the lateral migration and isolate water/proton from the aqueous electrolyte, thus eliminating dendrite and suppressing side reactions [11, 13]. Inorganic coatings with nanostructured morphology have been proven effective in promoting uniform Zn^2+^ deposition. However, rupture of the coating with brittleness and weak adhesion was unfavorable for long-term plating/stripping cycles [17–19]. On the contrary, organic polymer coatings with high adhesion and flexibility can adapt to changes in anode volume derived from long-term cycles. Besides, uniform Zn^2+^ distribution is regulated on the molecular scale via polar surface groups as abundant nucleation sites [20–22]. Nevertheless, stronger chemisorption toward Zn^2+^ rather than water molecules results in sluggish transport and reduced Zn^2+^ flux, leading to high polarization and instability of the anode interface [23].

For the stable plating/stripping process and low anodic polarization voltage, a delicate interface driving force with accelerated Zn^2+^ flux on the anode surface is particularly desirable. One effective method is to regulate the interface electric field through the dielectric interface that serves as a build-in driving force [24–26]. Dipoles are spontaneously polarized and alternately exhibit positively and negatively charged surfaces, corresponding to the charging and discharging process [25, 26]. The charged surface can achieve low anodic polarization and high rate performance by dynamically accelerating ion transport as well as adsorbing ions and homogenizing ion flux [27]. Focusing on the interface driving force via an adaptable electrical field has tremendous significance in terms of speed and uniformity to ions transport, but remains few alternatives.

Herein, we present amphoteric charged coatings based on silk fibroin (SF) with ionized side/end chains (-NH_2_ and -COOH) that stabilize and expedite Zn^2+^ flux on the anode surface. The operando pH-controlled surface charges during plating/stripping process (plating, pH below 4.1: –NH_2_ + H^+^ ≥ –NH_3_^+^; stripping, pH above 4.1: –COOH + –OH^−^ ≥ –COO^−^) permit the reversible adaptive electrical field direction. In addition to the interface electric field-controlled accelerated Zn^2+^ plating/stripping kinetics, the hydrophobic β-sheet served as a water barrier to suppress side reactions. Experimental analyses together with theoretical calculations suggested surface polar groups (–O=C–N–H) of SF facilitated the desolvation of [Zn(H_2_O)_6_]^2+^ and provided nucleation sites for uniform Zn deposition. With the assistance of SF coating, the symmetric cell shows a low voltage polarization (83 mV at 20 mA cm^−2^) and ultralong lifespans (1,500 h at 1 mA cm^−2^ and 500 h at 10 mA cm^−2^) with an exceptional cumulative capacity of 2.5 Ah cm^–2^. The full cell coupled with the Zn_x_V_2_O_5_·nH_2_O (ZnVO) cathode exhibits specific energy of ~ 270.5/150.6 Wh kg^−1^ (at 0.5/10 A g^−1^) with ~ 99.8% Coulombic efficiency (CE) and ~ 80.3% retention (at 5.0 A g^−1^) after 3,000 cycles. This work provides a novel perspective on expediting the kinetics of ionic migration for high-rate Zn anode.

## Experimental and Calculation

### Materials Preparation

Zn foil (> 99.99%) with a thickness of 100 µm was purchased from Saibo of Beijing. Bombyx mori silkworm cocoons were purchased from the Alibaba store. All the chemical reagents were purchased from Energy Chemical without further purification.

### Preparation of Zn-SF Anodes

Firstly, Bombyx mori SF was prepared according to the previous literature. Cocoons (5 g) were cut into pieces and boiled in 0.02 M Na_2_CO_3_ aqueous solution for 30 min, followed by washing thoroughly with distilled water and drying in the air at ambient temperature. Subsequently, 0.2 g CaCl_2_ was dissolved in anhydrous formic acid to obtain a homogeneous and transparent solution. 0.6 g SFs were added to the above solution under vigorous stirring until complete dissolution. Finally, the mixture solution was cast onto the polished zinc foil and dried overnight to prepare a solid SF coating. The dried coating was soaked in water to induce the formation of β-sheet and pores. To prepare the Cu-SF electrode, the mixture solution was cast onto the Cu foil, and the electrode was treated with water after drying overnight.

### Preparation of ZnVO Cathode

The Zn_x_V_2_O_5_·nH_2_O (ZnVO) was synthesized by hydrothermal reaction according to the reported literature [28]. Firstly, V_2_O_5_ (0.365 g, 2 mmol) was dissolved into 50 mL deionized water and stirred for 10 min under an ambient environment. And then, H_2_O_2_ (2 mL), PEG (800 mg), and Zn(NO_3_)_2_·6H_2_O (0.119 g, 0.4 mmol) were then added to the above solution with violent stirring for 30 min. The mixture solution was then transferred into a Teflon-lined autoclave (100 mL) and heated at 120 °C for 12 h. After cooling to room temperature, the solution was centrifuged (10,000 rpm per 3 min) and washed with deionized water and ethanol at least three times. Finally, the product was collected as a black-green powder after vacuum drying at 60 °C overnight.

### Material Characterizations

The field emission scanning electron microscopy (SEM) equipped with an energy dispersive spectrometer (EDS) was employed to characterize the morphology of SF coating and zinc anode after deposition (FEI Nova Nano SEM 450). The X-ray diffraction (XRD, D/max2500pc diffractometer with Cu Kα radiation) was applied to characterize the crystal structure of ZnVO, SF coating, and Zn anode before/after Zn deposition. X-ray photoelectron spectroscopy (XPS, Thermo ECSALAB 250 having the Al anode) was introduced to reveal the product before/after Zn deposition with C 1* s* peak calibration (284.8 eV). The Fourier transform infrared spectra (FT-IR) were carried out by a spectrometer (BRUKER VECTOR 22 Spectrometer) to obtain the silk structure of SF coating. The generated gas (mainly H_2_) was quantitatively detected by gas chromatography (SHIMADZU GC-2014) with a thermal conductivity detector (TCD).

### Preparation of Zn (Zn-SF)-ZnVO Full Cells

The ZnVO powder, acetylene black, and poly(tetrafluoroethylene) (PTFE) were mixed with mass ratios of 7:2:1 to prepare the self-supporting membrane. The membrane was compacted to the stainless steel (SS) mesh and was cut into pieces (1 × 1 cm^2^) after vacuum drying at room temperature. The mass loadings were measured to be 2 mg cm^−2^. The cathode was assembled in a CR2025 coin cell with Zn foils (Zn-SF) as the anode. The glass fiber filters (GF/C, Whatman) and 2 M ZnSO_4_ were used as separators and electrolytes, respectively.

### Coating Surface Charge

To investigate the stability and surface charge of SF coatings, SF in formic acid was dried, centrifuged, milled, and resuspended in the 2 M ZnSO_4_ solution at different pH. The surface charges of the suspensions at different pH values were measured using a Malvern Zetasizer (Nano series) under zeta mode.

### Electrochemical Measurements

The exchange current density was measured in 2 M ZnSO_4_ electrolyte by three-electrode configuration, with Zn (Zn-SF) as the working electrode, Zn foil as the counter electrode, and Zn wire as the reference electrode. Using the same three-electrode configuration, hydrogen evolution reaction (HER) activity was evaluated by linear sweep voltammetry (LSV) in 2 M Na_2_SO_4_ electrolyte. The nucleation overpotential and chronoamperometry curves were measured in 2 M ZnSO_4_ electrolyte using the three-electrode configuration with Zn wire as a reference electrode. All three-electrode configurations were carried out on the electrochemical workstation CHI 660e. The stripping/plating process of zinc anode was detected by galvanostatic cycling on LAND battery testers, including different C-rate, utilization, and cycling stability. Electrochemical impedance spectroscopy (EIS) of symmetrical cells was recorded by electrochemical workstation CHI 660e with a frequency range from 10^6^ to 10^–2^ Hz. Cyclic voltammetry (CV), self-discharging, and EIS curves of the full cells were conducted on electrochemical workstation CHI 660e. The rate performance and cycling stability were tested by galvanostatic charging–discharging (GCD) methods on the LAND battery testers. The Coulombic efficiency of asymmetric batteries and full cells was obtained from LAND battery testers using GCD methods.

The pH of the 2 M ZnSO_4_ electrolyte near the anode during the stripping/plating process was monitored using two-configuration in a 50-mL H-type cell with the same Zn (Zn–SF) as the working electrode and another Zn (Zn-SF) as counter and reference electrodes. The H-type cell was connected to gas chromatography to detect the possible hydrogen.

### Molecular Dynamics (MD) and Density Functional Theory (DFT) Calculation

MD and DFT computations were carried out according to the reported literature [29]. GROMACS [30] (version 5.1.4) program was performed for the molecular dynamics simulations using Amber force field [31] and conformations for the density functional theory (DFT) calculation. A simple point SPC/E model [32] was used to describe water molecules, and three systems were constructed in this work. One was 6 ZnSO_4_ with 100 water molecules in a 2 × 2 × 2 nm^3^ simulation box with periodic boundary conditions. The other one was an SF (GSGAGA) with 100 water molecules in the same simulation box. The last one was an SF with 6 ZnSO_4_ solvated by 100 water. The steep descent method was used to minimize the energy of each system, and molecule dynamics were carried out under NPT ensemble at 300 K/1 atm with 20 ns for three systems. The bond lengths of each component were constrained by LINCS algorithm [33]. The V-rescale thermostat algorithm was used to keep the temperature [34]. The cut-off distance for the Lennard-Jones and electrostatic interaction was set as 1.0 nm in the simulation. Long-range electrostatics were calculated by using particle mesh Ewald (PME) method [35] with a Fourier grid spacing of 1.6 Å.

For the DFT calculation, the structure of Zn^2+^, SF, and water molecules was selected from molecule dynamics. The structure optimization was performed with ωB97XD/def2-SVP [36] level based on the Gaussian 16 package. And the single-point energies of complexes were done at the same level after optimization. The binding energy (Eb) of Zn^2+^-H_2_O, Zn^2+^-SF, and SF-H_2_O is calculated by Eq. (1):

1 $${E}_{b}={E}_{total}-{(E}_{1}+{E}_{2})$$

where *E*_*b*_ was the total energy of the complex system, and *(E*_*1*_ + *E*_*2*_*)* was the sum of the energy of each component.

### Electric/ion Field Simulation

The electric field and Zn^2+^ concentration field distributions at the anode/electrolyte interface were simulated using a simplified 2D model based on COMSOL Multiphysics 6.0 software. In this model, the length of the two Zn electrodes was 5.0 μm, and the thickness of the Zn electrodes was 0.3 μm, with a distance of 3.2 μm between them. The surface morphologies of bare Zn and Zn-SF in this simulation were based on the SEM observation. The voltage hysteresis from symmetric cells was the same and set as 28 mV with 1 mA cm^−2^ where the anodic potential was a constant of 0. The surface charge density of SF coating was 10^–5^ C m^−2^. Coating without charge was also simulated using the same methods. The operating temperature was 298 K. The initial ion flux for the ZnSO_4_ electrolyte was 2 M.

## Results and Discussion

### Mechanism of Zn^2+^ Transport at Coating Interface

**Fig. 1:**
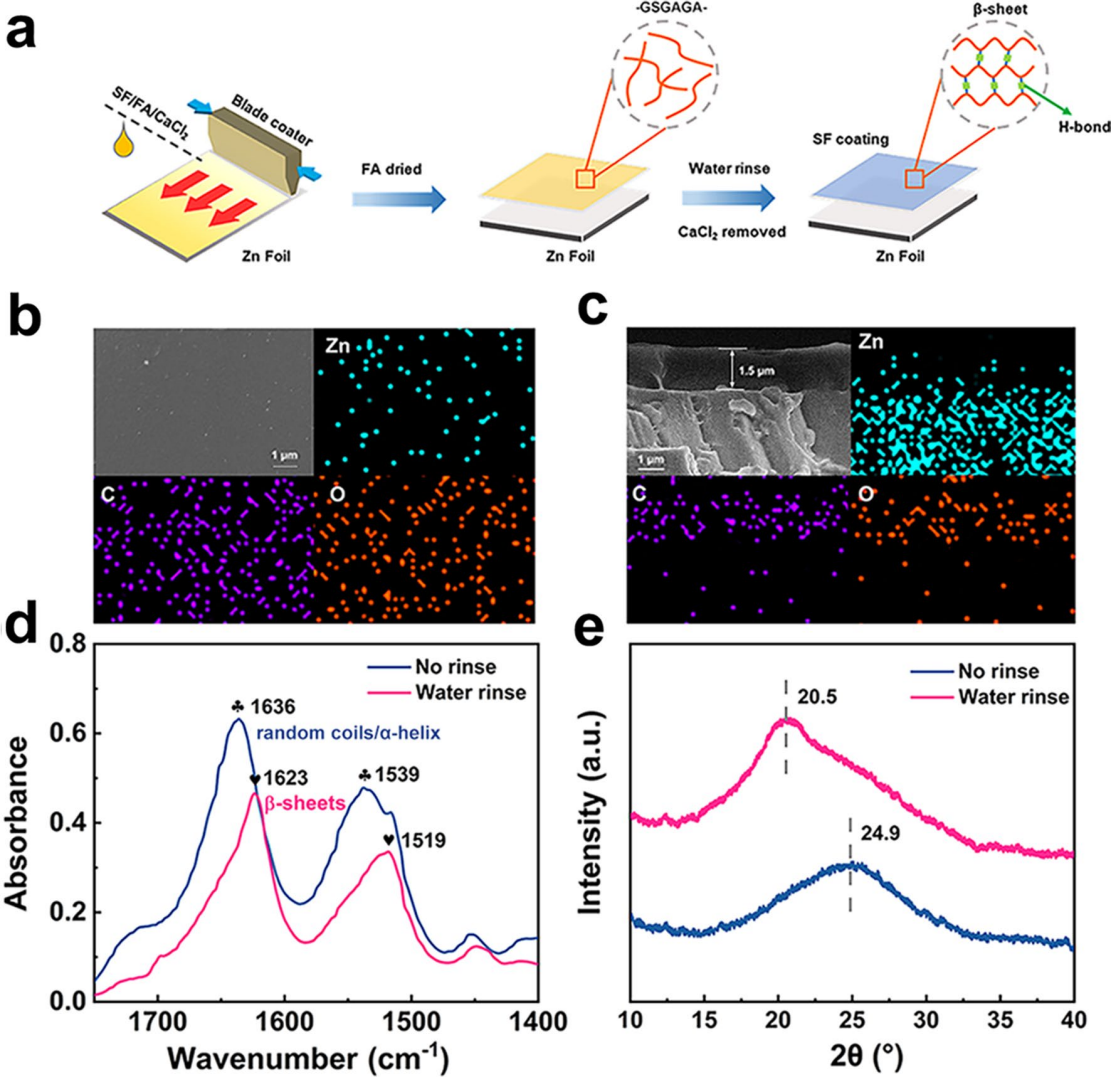
Preparation and characterization of Zn-SF anode. **a** Preparation of Zn anode with SF coating. **b** Surface and **c** cross section SEM images of Zn-SF anode after a water rinse. **d** FT-IR spectra and **e** XRD patterns of SF coating before/after water rinse

Figure 1a illustrates the preparation of the SF-coated Zn foil. SF aqueous solution was cast onto the Zn surface by the dissolution–regeneration process. After water rinsing, CaCl_2_ was removed and silk fibroin molecular chains were stabilized by a large number of intrachain and interchain hydrogen bonds, forming hydrophobic, oriented regions with a high concentration of regular β-sheets that only swell (rather than dissolve) in water [37, 38]. SEM and EDS exhibited smooth coating surfaces (Fig. 1b) with an ultrathin thickness of 1.5 μm (Fig. 1c). In the FT-IR spectrum (Fig. 1d), the characteristic peaks of α-helix and random coil appeared around 1636 and 1539 cm^−1^. The characteristic peaks of β-sheet appeared around 1623 and 1519 cm^−1^ [38–40]. Water treatment promoted the transition from α-helix/random coil to β-sheet. In the XRD pattern (Fig. 1e), the β-sheet was also confirmed by the peak at 20.5° [38]. Regenerated silk fibroin dissolved in some organic solvents by breaking intermolecular hydrogen bonds (β-sheet), forming random coil/α-helix structures. Further immersion in water revealed very regular and ordered amino acid repeating polypeptide fragment chains (such as GSGAGA chains), which can be tightly packed through hydrogen bonds and form antiparallel β-sheet nanocrystals [38, 41, 42]. This structural transition to crystalline β-sheet anchored water molecules and prevented water penetration to the Zn anode. At the same time, the hydrophobicity of the β-sheet was also beneficial in inhibiting the side reaction of Zn corrosion. As a result, the contact angle increased from 70.5° (Zn-SF without water rinsing) to 100° (Zn-SF with water rinsing) compared to bare Zn (87°) (Fig. S1). Additionally, although lower than pure 2 M ZnSO_4_ electrolyte (57 mS cm^−1^, Fig. S2a), SF film showed ideal ionic conductivity (2 mS cm^−1^, Fig. S2b) prior to the previous coating reports [21, 43].

**Fig. 2:**
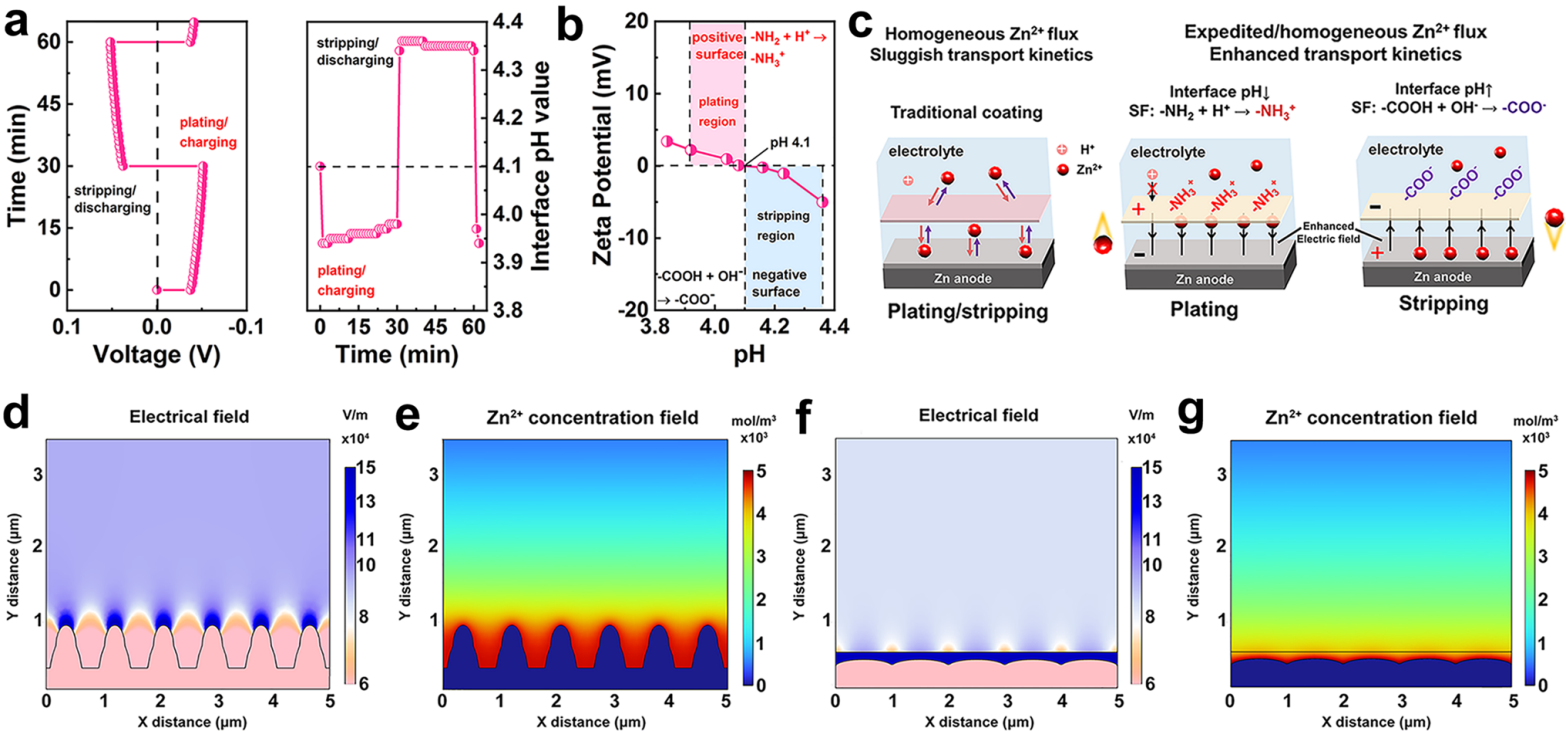
Reversible electrical field controlled by interface pH. **a** Real-time interface pH with the plating/stripping process of the Zn-SF anode at 5 mA cm^−2^. **b** Zeta potential of SF in the 2 M ZnSO_4_ electrolyte with different pH. **c** Mechanism of the build-in electrical field with dynamic interface pH. **d-g** Simulated electric field and Zn.^2+^ concentration field distributions on bare Zn (**d, e**) and Zn-SF (**f, g**)

A homemade operando pH detection configuration was constructed to monitor the pH evolution for the solid interface during the stripping/plating process (Fig. 2a). During the plating process of the Zn-SF symmetric cell, the interface pH value rapidly dropped until a stabilized value of 3.92, attributed to the suppressed hydrogen evolution reaction (HER) by the SF coating. During the stripping process, the interface pH value was maintained at 4.36, attributed to the slow consumption of OH^−^ [44]. In contrast, the interface pH values of bare Zn (Fig. S3) displayed similar trends but dramatic changes due to direct exposure to water/proton and severe HER, leading to rapid cell failure. To examine the surface charge of SF in 2 M ZnSO_4_ electrolyte, we measured zeta potential across the pH range of 3.84–4.36 (Fig. 2b). The net-zero surface charge of SF (isoelectric point of) was around 4.10. SF surface showed negative zeta potential above pH 4.1 and positive zeta potential below pH 4.1, proving reversible negative/positive charges on the SF surface under different pH [45]. Thus, a reversible interface electric field and build-in interface driving force could be established by regulating the coating surface charge endowed by dynamically changed interface pH during the stripping/plating process (Fig. 2c). According to Fick’s law and Poisson's equation, the diffusion flux of ions was relative to the concentration gradient of ions and the distribution of the electric field [27]. The liquid-phase ion transport (57 mS cm^−1^) in the electrolyte was much faster than the solid-state diffusion in the coating (2 mS cm^−1^), so the solid-state ion transport inside the coating could be crucial for anodic polarization (Zn anode with coating usually had higher overpotential than those of bare Zn due to stronger chemisorption of coating toward Zn^2+^) [11, 23, 46]. Therefore, an enhanced interface electric field by SF coating could expedite Zn2 + flux and further homogenize the ion transport to form the even ion flux, finally realizing the enhanced transport kinetics [47].

To further illustrate the role of the charged SF coating in regulating the interfacial electric field and concentration field, a finite element simulation based on COMSOL Multiphysics was carried out. Figure 2d-e showed the negative effects of sharp dendrites of a bare Zn electrode. Inhomogeneous ion distribution and enhanced field intensity on the protuberances resulted in the growing tips. Compared to neutral coating (Fig. S4a-b), Zn-SF displayed homogeneous and enhanced interface electrical fields, which led to expedited Zn^2+^ flux (Fig. 2f-g) through interface driving force.

To reveal the influence of the reversible electrical field, the stripping/plating overpotentials of Zn-SF were measured in a three-electrode configuration with different pH electrolytes (2 M ZnSO_4_). During the plating process, interface pH below the isoelectric point enabled a positive charge surface, leading to an accelerated Zn^2+^ deposition with lower overpotentials (Fig. S4c). During the stripping process (Fig. S4d), interface pH above the isoelectric point attracted Zn^2+^ migration to SF coating due to negative charges on the coating, enabling a stripping process. Considering overpotentials during the reversible process, coatings with single charges can only contribute to stripping or plating, whereas the SF coating with reversible amphoteric charges accelerated Zn^2+^ migration in both processes.

### Electrochemical Properties of Symmetric Cells

**Fig. 3:**
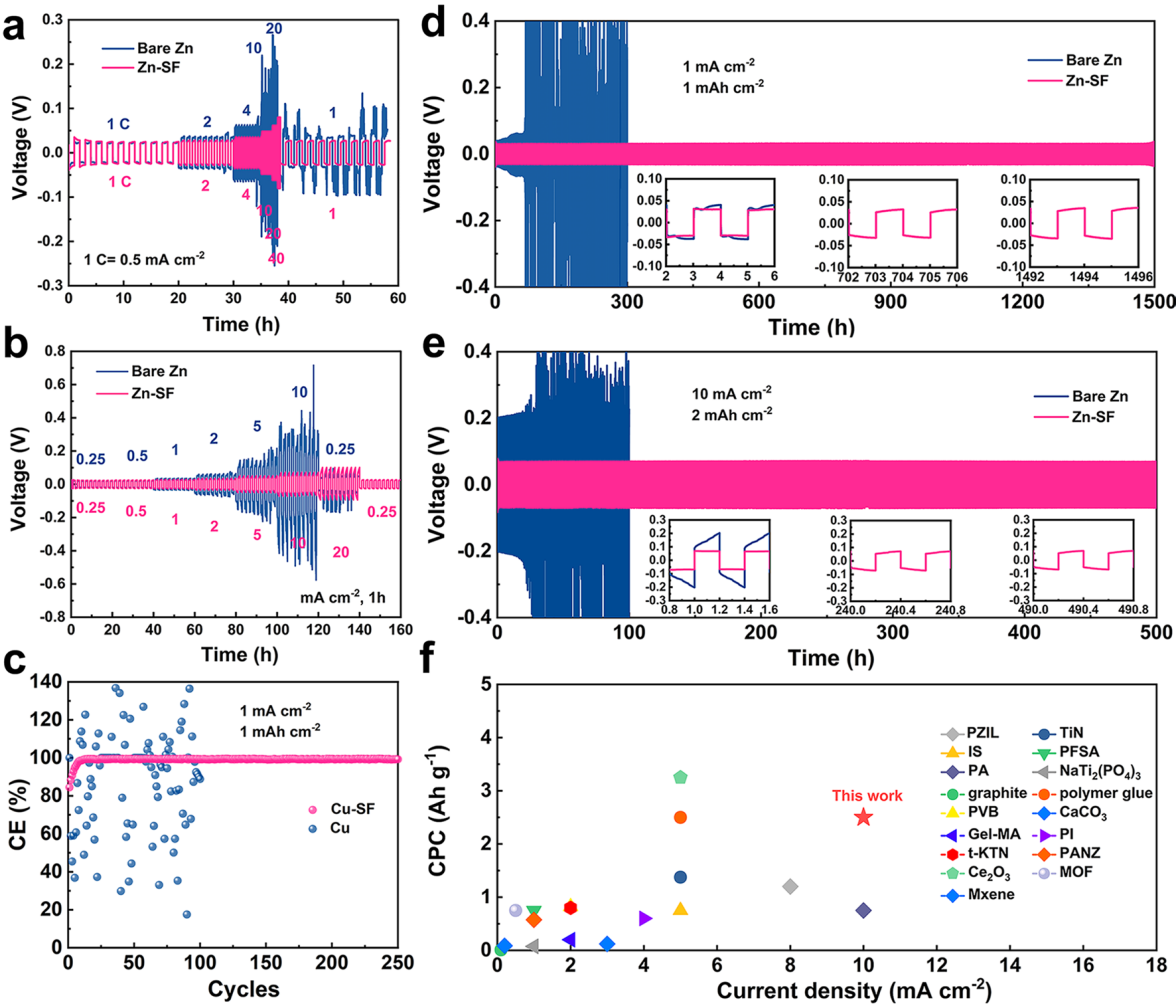
Electrochemical performances of symmetric cells with bare Zn and Zn-SF electrodes. **a** Rate performance of symmetric cells at current densities from 0.5 to 20.0 mA cm^−2^. **b** Plating/stripping process with different depths (0.25 to 20 mAh cm^−2^) **c** CE of the two anodes at 1 mA cm^–2^/1 mAh cm^–2^. **d, e** Voltage profiles of bare Zn and Zn-SF anodes at (**d**) 1 mA cm^–2^/1 mAh cm^–2^ and (**e**) 10 mA cm^–2^/2 mAh cm.^–2^. **f** Comparison of cumulative capacity and current density of Zn-SF with other reported Zn anodes with various coating layers (PZIL [13], TiN [17], IS [20], PFSA [21], PA [11], NaTi_2_(PO_4_)_3_ [50], graphite [51],polymer glue [52], PVB [53], CaCO_3_ [18], Gel-MA [54], PI [22], t-KTN [26], PANZ [55], Ce_2_O_3_ [24], MOF [56], MXene [19])

Thanks to the electric field acceleration, the Zn-SF anode in symmetric batteries possessed a higher Zn^2+^ transference number (0.77) than bare Zn (0.54) (Fig. S5a-b). It also exhibited excellent rate performance from 1C (0.50 mA cm^−2^) to 40C with stable voltage hysteresis of 83 mV, indicating the tolerance for high current density (Fig. 3a). Compared to the reports using silk fibroin as an electrolyte additive, the electric field generated additional driving force. Therefore, the Zn-SF anodes showed a lower voltage hysteresis than those of electrolyte additive-based Zn anodes [29, 46]. In contrast, the bare Zn anode displayed similar voltage at the low rate but rapid growth (206 mV, 40C) with unstable stripping/plating at the high rate. The stripping/plating process became difficult for the bare Zn anode due to the block of ion transport channels by passivation products (ZHS) [5, 48]. The depth of discharge (DOD) for anodes was further investigated using different capacities. The amount of sites involving the reaction on the zinc surface steadily decreased as the cumulative capacity rose, making deposition and stripping more difficult. Therefore, both anodes exhibited increased voltage hysteresis as DOD increased compared to those of rate performance (Fig. 3b) [49]. The bare Zn displayed an unstable voltage and could not recover due to irreversible transformation and accumulation of side products.

On the contrary, the Zn-SF anode displayed a smaller polarization and stable voltage hysteresis even at 20 mAh cm^−2^ (33% of DOD), proving the excellent reversibility of the modified Zn anode. Moreover, the reversibility of Zn plating/stripping was evaluated by Cu(Cu-SF)//Zn asymmetric cells (Figs. 3c and S6). CE of bare Cu//Zn cell showed a drastic fluctuation at 1 mAh cm^−2^ (1 mA cm^−2^), indicating the low utilization of active Zn caused by side reactions as well as the formation of ZHS [28]. In contrast, the high reversibility of Cu-SF can be further observed by the stable voltage curves (1st, 5th, 10th, 50th, and 100th cycle) with an average CE of 99.2% over 250 h. Through stabilizing the Zn anode, SF coating was able to suppress Zn dendrites and endow ultralong anode lifespan. The bare Zn exhibited severe voltage fluctuations with a short circuit after 300 h at 1 mAh cm^−2^ (1 mA cm^−2^) cycling, while the polarization voltage of the Zn-SF anode retained 32 mV up to 1,500 h with a slight increase (Fig. 3d). Even when the cutoff capacity was amplified to 2 mAh cm^−2^ at a high current density of 10 mA cm^−2^ (Fig. 3e), excellent cycle endurance up to 500 h was recorded for the Zn-SF anode, demonstrating a more stable anode than pure Zn anode (200 h with wildly fluctuating voltage).

The EIS results of symmetric cells can also confirm the excellent cycle stability after various cycles (Fig. S7). After 100 cycles, the Zn-SF//Zn-SF cell delivered lower charge transfer resistance (Rct) of 200 Ω than that of the Zn//Zn cell (920 Ω). The performance of Zn-SF was also compared with the art-of-the-state Zn anodes modified by other organic/inorganic coatings (Figs. 3f and S8). Owing to the interface electrical field, the Zn-SF anode showed lower voltage polarizations and max current density up to 20 mA cm^−2^, which displayed excellent rate performance. The Zn-SF anode also had an impressive cumulative capacity at high rates (2.5 Ah cm^−2^ at 10 mA cm^−2^), among the highest in the coating-modified Zn anodes.

**Fig. 4:**
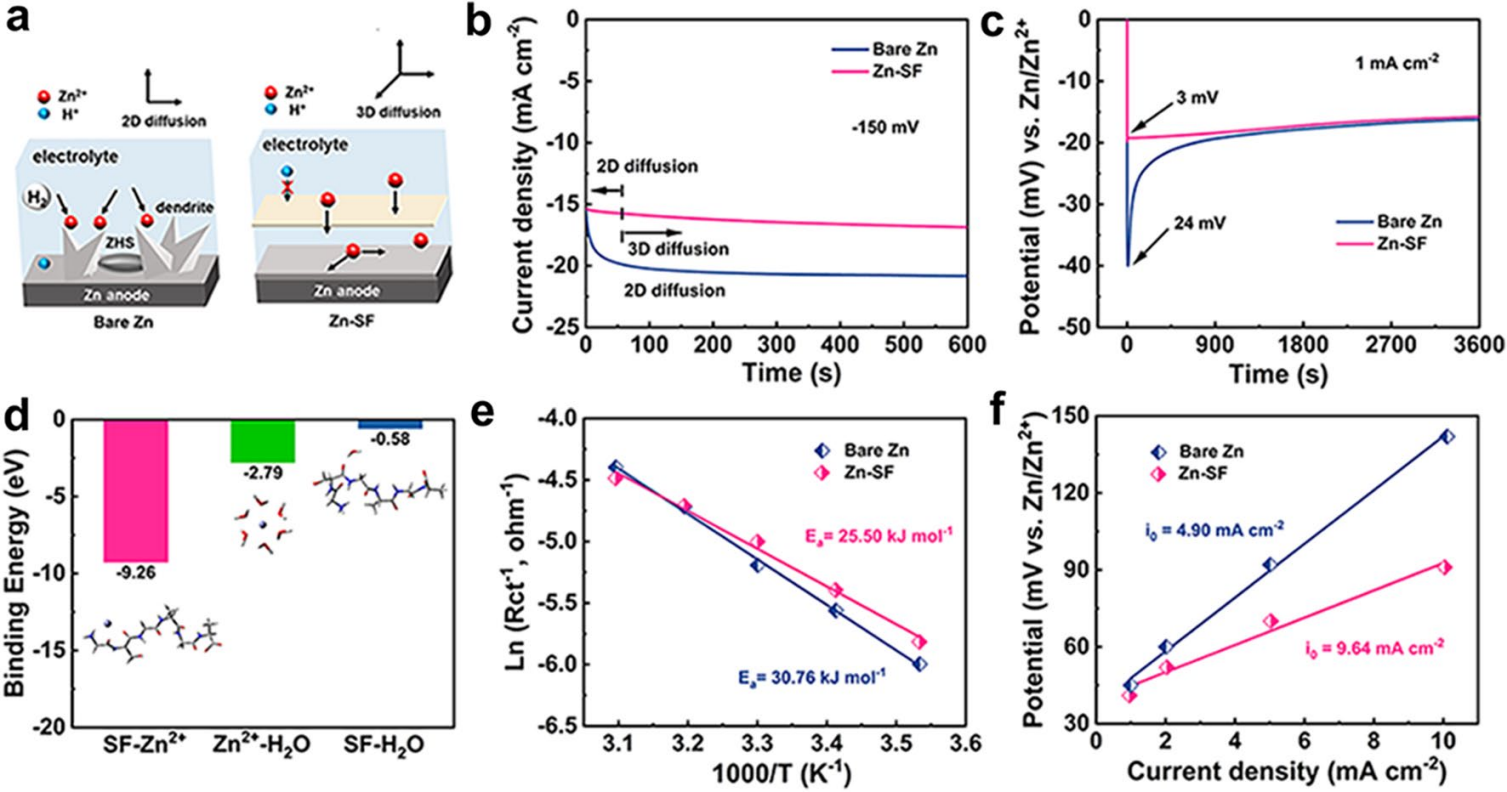
Investigation of SF-assisted deposition and desolvation behavior. **a** Schematic diagram demonstrating the SF-assisted uniform deposition and suppressed H_2_. **b** Chronoamperometry curves at an overpotential of -150 mV. **c** Nucleation overpotentials of Zn electrodeposition. **d** Binding energy between Zn^2+^ and various molecules (H_2_O and GSGAGA) from density functional theory calculations. **e** Desolvation activation energies fitted by Arrhenius curves. **f** Exchange current densities from Tafel curves at 1 mV s.^−1^

Apart from electric field acceleration, the interface was beneficial to uniform Zn growth by regulating the diffusion behavior of ions. The chronoamperometry test was applied in a three-electrode configuration with an overpotential of -150 mV. The current of the bare Zn anode continued to increase during 600 s, suggesting a random 2D diffusion on the tips (**Fig. ****4****a-b**). In comparison, the Zn-SF anode only delivered rapidly increased current for initial nucleation and maintained a steady current with restricted 3D diffusion during Zn growth [11]. Nucleation behaviors were evaluated by galvanostatic deposition at 1 mA cm^−2^ (**Fig. ****4****c**). It is found that after constructing the SF interface, the nucleation overpotential decreased from 24 to 3 mV, attributed to the nucleation sites by abundant polar groups. SEM was also measured at 5 mA cm^−2^ (10, 30, and 60 min) to observe the deposition morphology of bare Zn and Zn-SF anode (Fig. S9). Due to the tip effect, the zinc ions continuously aggregated to form dendrites, and with the increase in deposition time, the dendrites further grew, accompanied by the HER reaction, resulting in the massive generation of the by-product ZHS. On the contrary, owing to the abundant nucleation sites of the coating, more crystal grains were formed on the zinc surface at the initial stage. With the increase in deposition time, zinc ions grow parallel to the anode surface, and the grains were connected with each other, and there finally formed a dense deposition layer.

Furthermore, DFT calculations were implemented to explore the role of the polar groups on the SF layer in regulating Zn deposition. SF was truncated into the GSGAGA segment model (Fig. S10). Figure 4d shows the binding energy between SF coating and Zn^2+^. The binding energy of GSGAGA-Zn^2+^ (−9.26 eV) is higher than that of Zn^2+^-H_2_O (−2.79 eV) and GSGAGA-H_2_O (-0.58 eV), indicating the capture of Zn^2+^ by SF coating and accelerated desolvation of Zn(H_2_O)_6_^2+^ [13]. The desolvation energy of Zn^2+^ was also evaluated in the symmetric cell associated with charge transfer resistance (*R*_ct_) (Figs. 4e and S11a-b) under different temperatures (10–50 °C) [57]. The linear fitting results showed that desolvation activation energy (*E*_a_) decreased from 30.76 (Zn) to 25.50 (Zn-SF) kJ mol^−1^ after introducing the SF coating interface, suggesting the lower desolvation energy barrier endowed by SF. Moreover, Zn deposition kinetics was further estimated based on the exchange current density fitted by Tafel curves (Figs. 4f and S11c) [58]. The exchange current density of the Zn-SF anode (9.64 mA cm^−2^) was higher than that of the bare Zn anode (4.90 mA cm^−2^), indicating a faster electrochemical reduction rate of Zn^2+^ with SF-assisted desolvation.

**Fig. 5:**
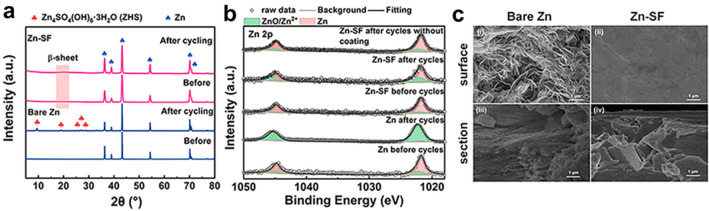
Structure characterization of bare Zn and Zn-SF electrodes before/after long-term cycles. **a** XRD patterns of bare Zn and Zn-SF anodes before/after cycles (Zn, PDF#04–0831; ZHS, PDF#39–0689). **b** XPS data of Zn 2*p* spectrum of two anodes before/after cycles. **c, d** SEM images of bare Zn (**c, i/iii**) and Zn-SF (**d, ii/iv**) anodes after cycles

XRD detected the formation of ZHS (PDF#39-0689) on the Zn (PDF#04-0831) surface after cycles, whereas ZHS was negligible on the Zn-SF surface (Fig. 5a). The suppression of by-products was studied in 2 M Na_2_SO_4_ (pH 4.1) by the linear sweep voltammetry (LSV). As shown in Fig. S12a-c, the Zn-SF electrode exhibited a higher overpotential and Tafel slope (900 mV and 336 mV dec^−1^) than the bare Zn (780 mV and 199 mV dec^−1^), reflecting the hydrogen suppression. Additionally, chemical compositions on the anode surface were examined by XPS. The Zn 2*p* spectrum in Fig. 5b showed many Zn (II) species (1045.3 and 1022.3 eV) for cycled Zn, corresponding to the mass formation of ZHS [15, 52]. The initial N, O, and S elements of Zn-SF electrodes before cycles (Fig. S13a-c) can be attributed to the SF coating [59]. After cycles, a weak Zn (II) signal and unchanged S/O elements were detected on the cycled Zn-SF surface, indicating few formations of ZHS. SEM images of the cycled Zn exhibited a rough surface/section with many sharp dendrites and passivation products (Fig. 5c(i, iii)), resulting in pierced separators and short circuits. On the contrary, a highly flat and dense surface/section was observed on the Zn-SF electrodes after cycles (Fig. 5c(ii, iv)). This well-evidenced the good interface stability and dendrite suppression capability by SF coating.

### Electrochemical Performance of the Full Cells

**Fig. 6:**
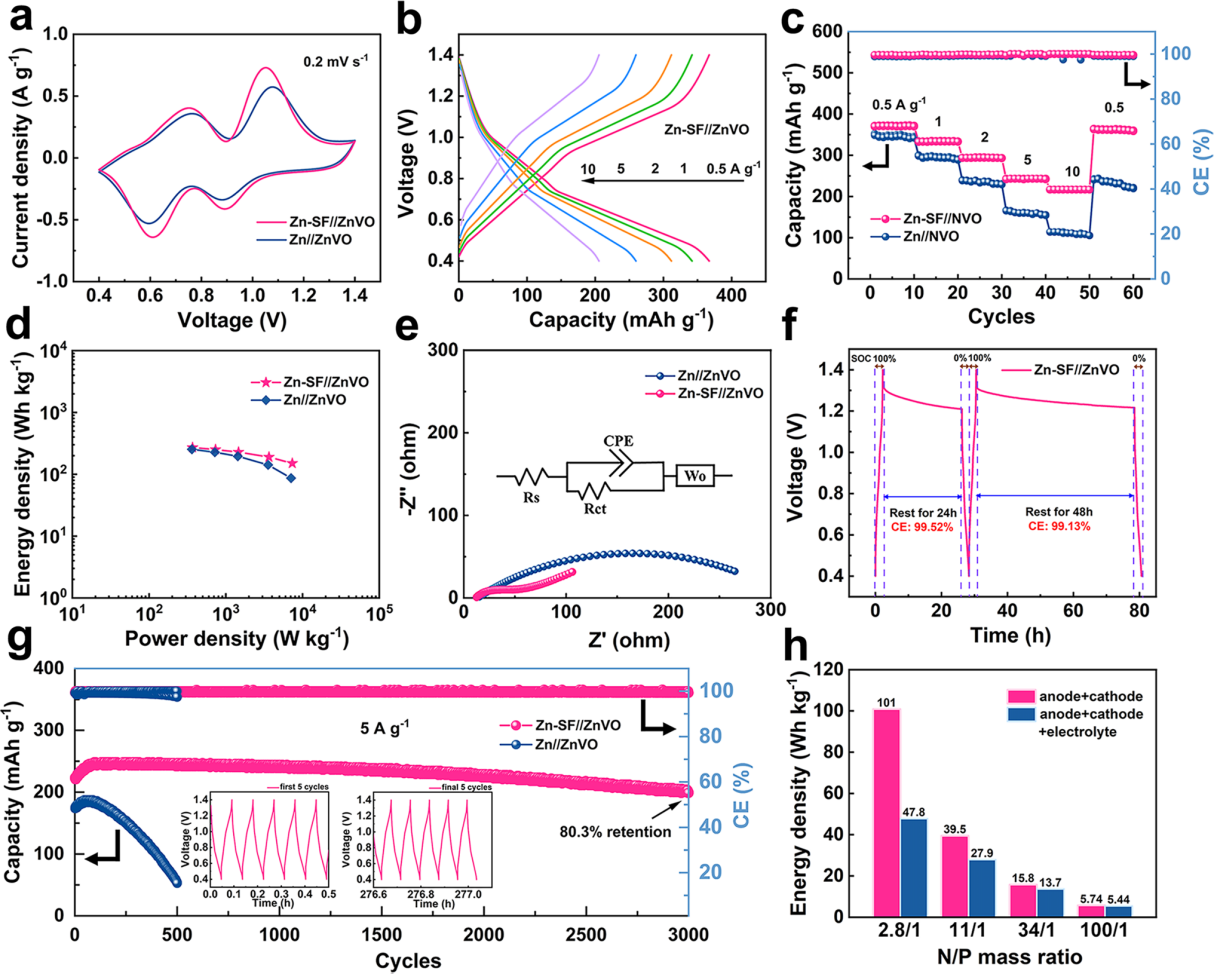
Electrochemical properties of full cells based on bare Zn and Zn-SF anode. **a** CV curves for the Zn//ZnVO and Zn-SF//ZnVO cells at a scan rate of 0.2 mV s^−1^. **b** Charging/discharging curves of Zn-SF//ZnVO cell at various current densities. **c** Rate performance of two cells. **d** Energy/power density of two full cells based on the mass of the cathode. **e** Comparison of two full cells in EIS results. **f** Evaluation of self-discharging level for Zn-SF//ZnVO cell rested at 100% stage of charge for 24 and 48 h. **g** Cycle stability of two cells at 5 A g^−1^. **h** Energy density values of Zn-SF//ZnVO cell based on the mass of anode + cathode/anode + cathode + electrolyte (5 mg electrolyte) under various N/P ratios

Full cells using ZnVO (XRD, Fig. S14a) as cathode were further constructed to verify the application of the Zn-SF anode. As Fig. 6a shows, cyclic voltammogram (CV) curves of the Zn//ZnVO and Zn-SF//ZnVO revealed two pairs of characteristic peaks (V^4+^/V^3+^ and V^5+^/V^4+^) at a scan rate of 0.2 mV s^−1^ [28]. Zn-SF//ZnVO cells exhibited higher current density and lower voltage gaps than Zn//ZnVO, representing decreased electrochemical polarization. Meanwhile, the Zn-SF//ZnVO delivered an excellent rate capability of 362 mAh g^−1^ at 0.5 A g^−1^ and 202 mAh g^−1^ at 10 A g^−1^, while the capacity of the Zn//ZnVO cell showed a lower specific capacity (350 mAh g^−1^ at 0.5 A g^−1^) and significantly declined capacity (120 mAh g^−1^ at 10 A g^−1^), caused by aggravated HER and side effects at high current densities (Figs. 6b-c and S14b). The energy/power density of Zn-SF//ZnVO cells based on the cathode raised to 270.5/150.6 Wh kg^−1^ at 0.5/10 A g^−1^ (Fig. 6d). EIS revealed the lower *R*_ct_ (50 Ω) of the Zn-SF//ZnVO cell and confirmed the fast charge transfer capability (Fig. 6e), thus leading to the superior rate performance.

The self-discharging behaviors of the full cells were evaluated by calculating CE for the rest of 24/48 h after being fully charged (Fig. 6f and S14c). After pristine voltage drop, the Zn-SF//ZnVO cell can maintain a higher CE (99.5%/99.1%) than that of Zn-ZnVO (97.5%/92.7%), attributed to the elimination of parasitic reactions and energy dissipation process by the SF coating [60, 61]. The Zn-SF//ZnVO cell exhibited superior stability (Fig. 6g, Table S1), maintaining 80.3% of the highest capacity with stable CE near 100% after 3,000 cycles (at 5 A g^−1^). In comparison, the Zn//ZnVO cell achieved only 25% capacity retention, fully revealing the stabilizing effect of SF coating for the anode. It was worth noting that the specific capacity of the full cells increased at the initial stage, which resulted from activation processes of the cathode with the widened interlayer spacing and more active sites [28, 62–64]. When Zn-SF//ZnVO full cell operated at a low current density (300 mA g^−1^) in 80 cycles, consistent with other V-based cathodes [65–67]., cathode dissolution resulted in a loss of active substances, leading to the declined capacity for a short time (80% retention of maximum capacity) in Fig. S15. To further highlight the practical values, we decreased the mass of zinc foils from 90 to 2.5 mg with 2 mg cathode active materials (corresponding N/P = 100/1, 34/1, 11/1, 2.8/1) to optimize anode usage and promote battery energy/power density based on device mass (Fig. 6h) [68]. The output voltage of the Zn-SF//ZnVO cell reduced as the anode discharging depth increased, leading to higher voltage polarization (Fig. S14d). Nevertheless, the decreased device mass significantly impacted energy/power density improvement. The Zn-SF//ZnVO cell could obtain a maximum capacity of 311.2 mAh g^−1^ (corresponding to the optimal energy density of 101/47.8 Wh kg^−1^ based on the mass of electrode/electrode + electrolyte) when the N/P is 2.8/1 with 30% DOD of the Zn anode, whose performance reached the practical application standards.

## Conclusions

In conclusion, a bio-inspired silk fibroin coating was engineered to stabilize the Zn anode via the dissolution–regeneration process. The amphoteric charges controlled by interface pH changes enabled a reversible built-in electrical field to expedite Zn^2+^ flux, thus accelerating ion migration and reducing anodic polarization. Additionally, polar surface groups accelerated Zn^2+^ desolvation and promoted uniform distribution, effectively aiding in a dendrite-free deposition. With the assistance of SF coating, the modified anode can deliver a low voltage polarization (83 mV at 20 mA cm^−2^) and long cycling life of Zn plating/stripping (1,500 h at 1 mA cm^−2^ and 500 h at 10 mA cm^−2^) with an exceptional cumulative capacity of 2.5 Ah cm^–2^. More importantly, the Zn-SF//ZnVO cell realized specific energy of ~ 270.5/150.6 Wh kg^−1^ (0.5/10 A g^−1^) and ~ 80.3% retention (at 5.0 A g^−1^) after 3,000 cycles with ~ 99.8% Coulombic efficiency. This bio-inspired amphoteric charged protein can provide a new approach to engineering interface electrical fields for dendrite-free and high-rate aqueous Zn batteries.

## Supplementary Information

Below is the link to the electronic supplementary material.

Supplementary file1 (PDF 1660 kb)
