# Supplementary material for: Interface Reversible Electric Field Regulated by Amphoteric Charged Protein-Based Coating Toward High-Rate and Robust Zn Anode
Source: Nanomicro Lett. 2022 Nov 10;14:219. doi: 10.1007/s40820-022-00969-4 (PMC9649586; doi:10.1007/s40820-022-00969-4)
Supplement: Supplementary file 1 — Supplementary file1 (PDF 1660 kb) [file 40820_2022_969_MOESM1_ESM.pdf]

Supporting Information for

# Interface Reversible Electric Field Regulated by Amphoteric Charged Protein-Based Coating toward High-Rate and Robust Zn Anode

Meihua Zhu[1, #], Qing Ran[3, #], Houhou Huang[1], Yunfei Xie[1], Mengxiao Zhong[1], Geyu Lu[2], Fu-Quan Bai[1, *], Xing-You Lang[3, *], Xiaoteng Jia[2, *], Danming Chao[1, *]

[1] College of Chemistry, Jilin University, Changchun 130012, P. R. China

[2] State Key Laboratory of Integrated Optoelectronics, College of Electronic Science and Engineering, Jilin University, Changchun 130012, P. R. China

[3] Key Laboratory of Automobile Materials, Ministry of Education, School of Materials Science and Engineering, Jilin University, Changchun 130022, P. R. China

[#] Meihua Zhu and Qing Ran have contributed equally to this work.

* Corresponding authors. E-mail: chaodanming@jlu.edu.cn (Danming Chao), baifq@jlu.edu.cn (Fu-Quan Bai), xtjia@jlu.edu.cn (Xiaoteng Jia), and xylang@jlu.edu.cn (Xing-You Lang)

## Supplementary Figures and Tables

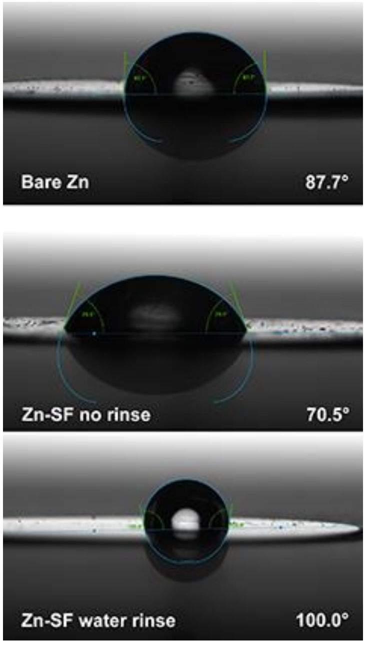


**Fig. S1** Images of the contact angle tests of 2 M $ZnSO_4$ on bare Zn and Zn-SF with/without water rinsing

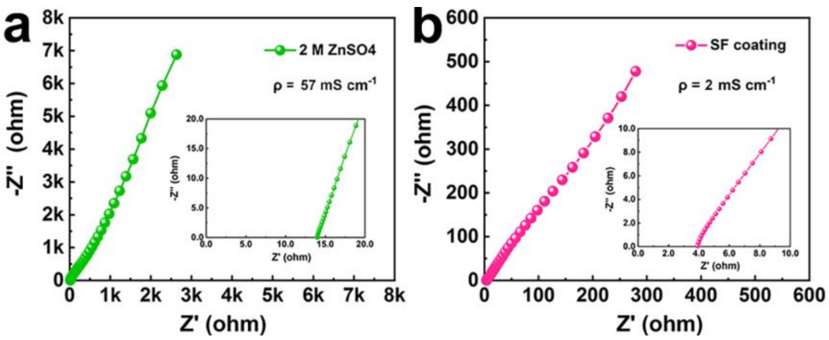


**Fig. S2** Ionic conductivity of the electrolyte (**a**) and SF coating (**b**) with electrolyte soaked by EIS results in the frequency range from $10^6$ Hz to $10^{-2}$ Hz

The ionic conductivity of the SF/electrolyte was tested by blocking electrode cells (SS//SF (or electrolyte) //SS) and calculated according to the Eq. S1:

$$\rho = \frac{l}{R \times A} \quad \text{(S1)}$$

where $R$ represented the resistance according to EIS measurement, $l$ represented the thickness of the SF (0.08 mm in Swagelok cell) or electrolyte (8 mm electrolyte in H-cell), and A (1 $cm^2$) was the area of contact between the stain steel and coating (or electrolyte).

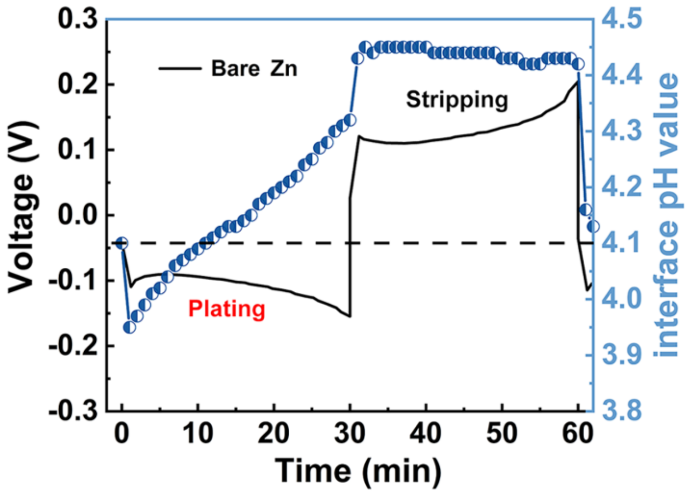


**Fig. S3** Real-time interface pH with the plating/stripping process of the Zn anode at 5 mA $cm^{-2}$

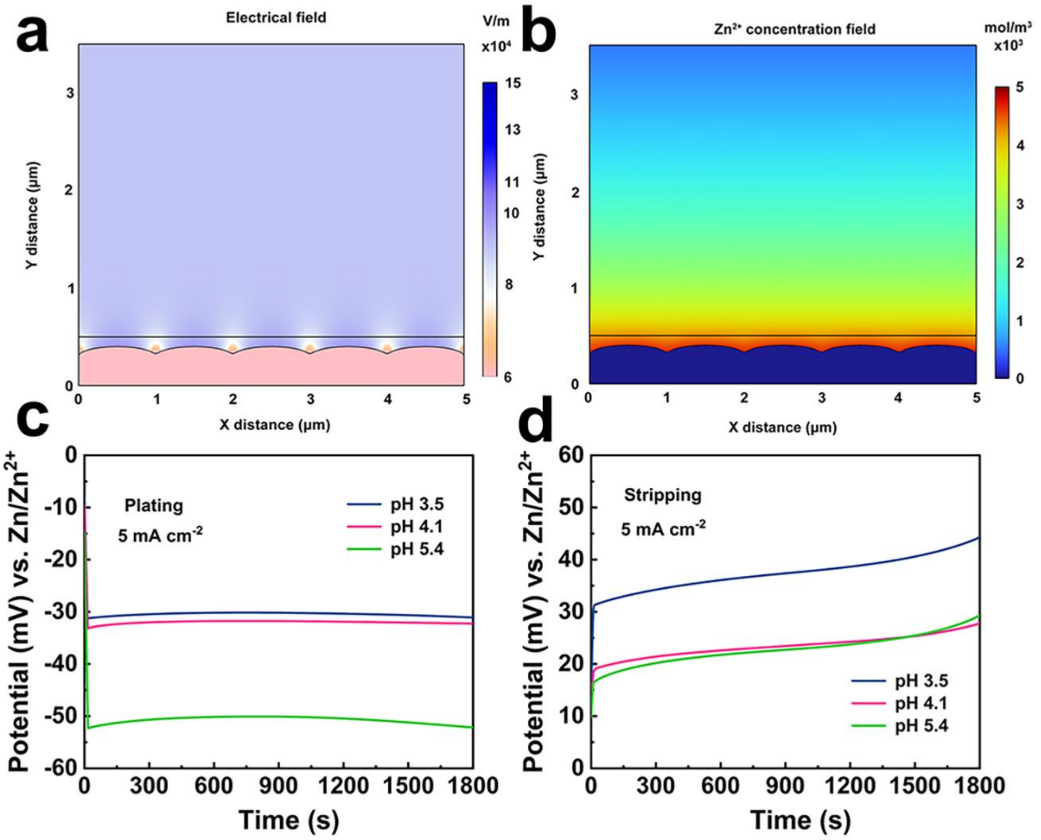


**Fig. S4** Simulated electric field (**a**) and $Zn^{2+}$ concentration field (**b**) distributions on Zn anode with natural coating. The plating (**c**) and stripping (**d**) overpotentials of Zn-SF anode with various charges in different pH electrolytes

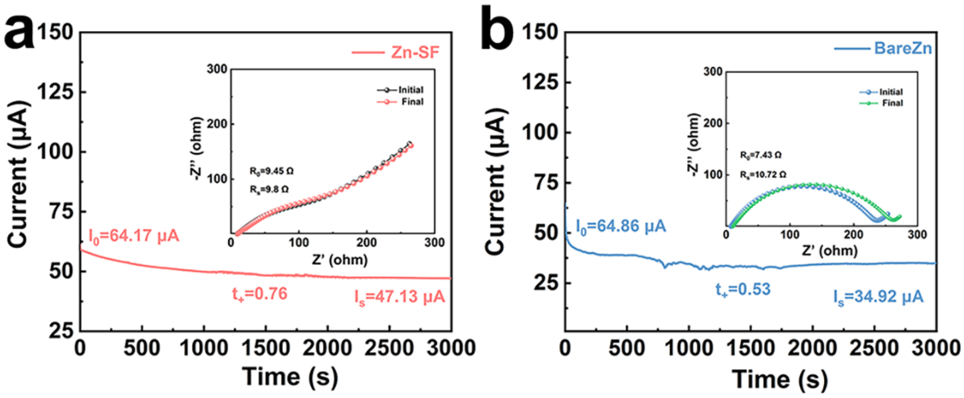


**Fig. S5** $Zn^{2+}$ transference number of Zn-SF (**a**) and bare Zn anode (**b**)

The ionic transference number was tested using a symmetric battery and calculated by the Eq. S2:

$$t_+ = \frac{I_S(\Delta V - I_0 R_0)}{I_0(\Delta V - I_S R_S)} \tag{S2}$$

where $\Delta V$ was the polarization voltage (15 mV); $I_0$ and $R_0$ were the initial current and resistance, respectively; and $I_s$ and $R_s$ were the steady-state current and resistance.

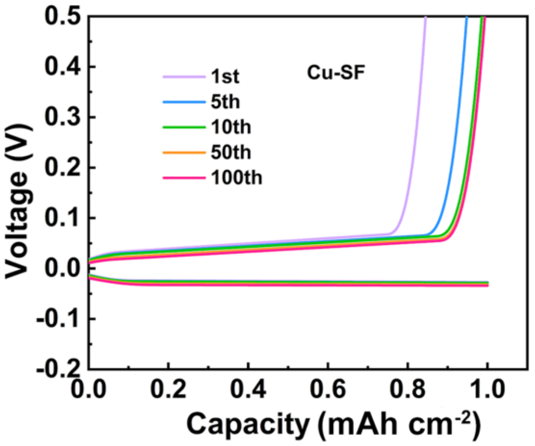


**Fig. S6** Voltage profiles of the Cu-SF//Zn cell at the 1st, 5th, 10th, 50th, and 100th cycles

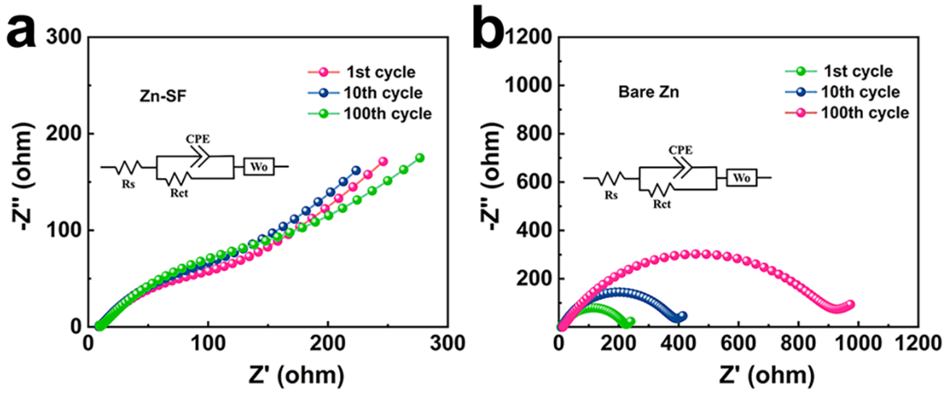


**Fig. S7** The electrochemical impedance of symmetric batteries with Zn-SF (**a**) and bare Zn (**b**) electrodes after cycles

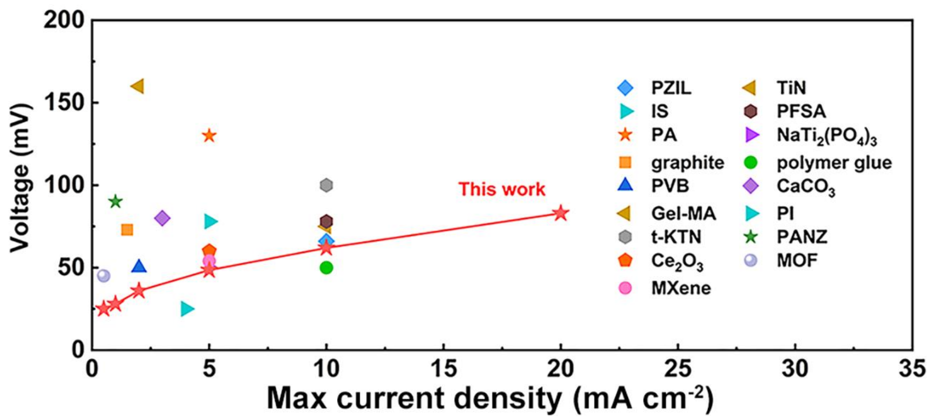


**Fig. S8** Comparison of voltage hysteresis and current density of Zn-SF with other advanced Zn anodes in the literature (PZIL [S1], TiN [S2], IS [S3], PFSA [S4], PA [S5], $NaTi_2(PO_4)_3$ [S6], graphite [S7], Polymer Glue [S8], PVB [S9], $CaCO_3$ [S10], Gel-MA [S11], PI [S12], t-KTN [S13], PANZ [S14], $Ce_2O_3$ [S15], MOF [S16], MXene [S17])

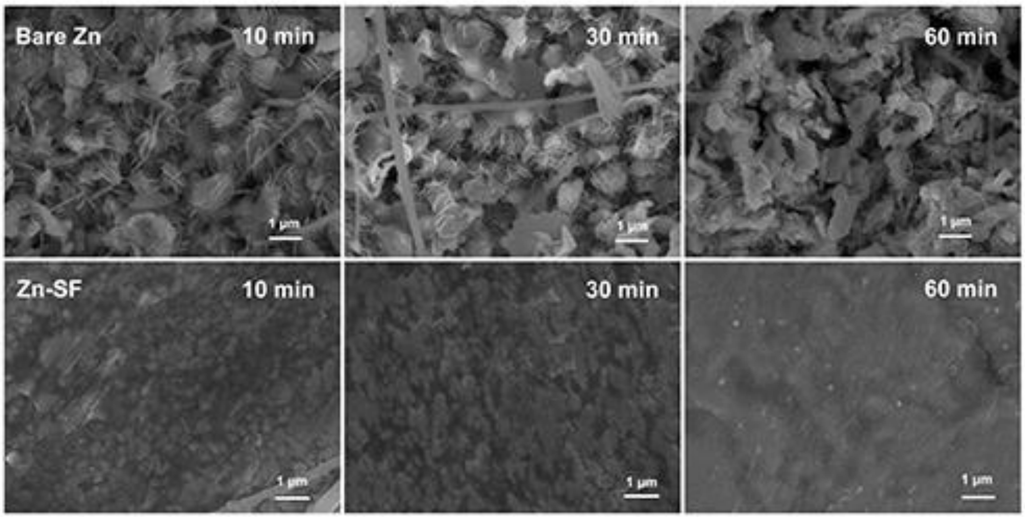


**Fig. S9** SEM images of deposition morphology of bare Zn and Zn-SF under 5 mA cm$^{-2}$ at 10, 30, 60 min

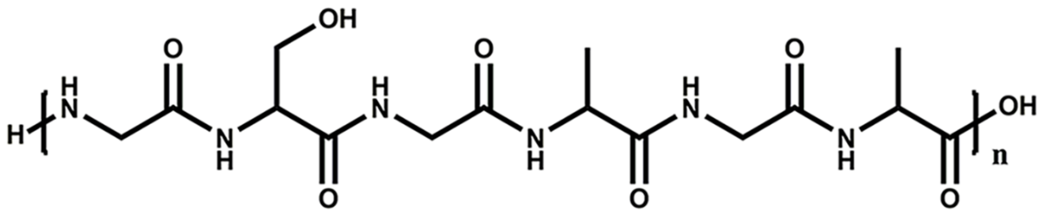

**Fig. S10** Chemical structure of GSGAGA segments in silk fibroin

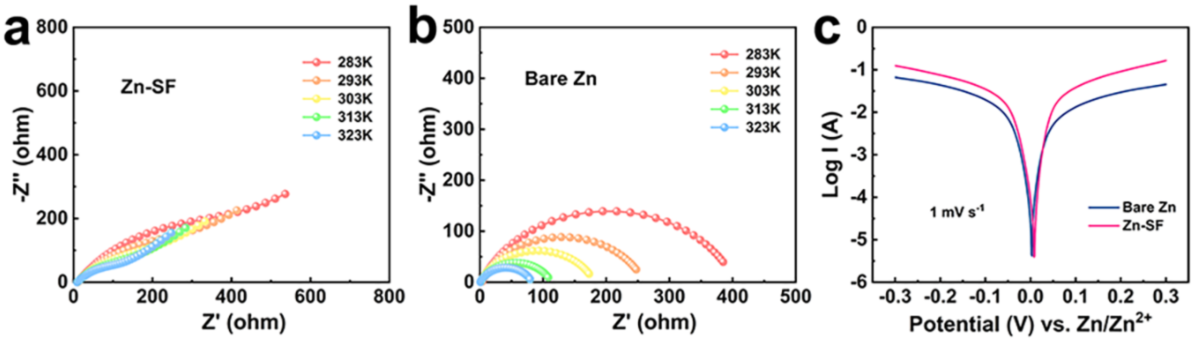


**Fig. S11** EIS data of Zn-SF//Zn-SF (**a**) and Zn//Zn (**b**) systematic cells under different temperatures. (**c**) Tafel plots of two electrodes at 1 mV $s^{-1}$

The activation (desolvation) energy ($E_a$) of the anode based on the symmetric cell was calculated according to Eq. S3:

$$\frac{1}{R_{ct}} = \mathrm{A} \times \exp\left(\frac{-E_a}{RT}\right) \tag{S3}$$

where *R* and *T* stood for the ideal gas constant and Kelvin temperature, respectively. The exchange current density ($i_0$) of the anode was calculated according to Eq. S4:

$$i = \frac{i_0 \times F \times \eta_{total}}{2 \times R \times T} \tag{S4}$$

where *i* and $\eta$ stood for the current density and total overpotential, respectively. *F*, *R*, and *T* represented the faradic constant, ideal gas constant, and Kelvin temperature, respectively.

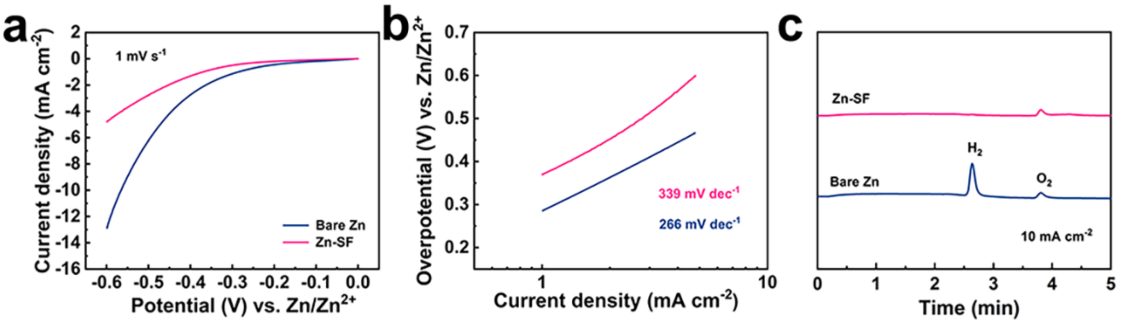


**Fig. S12** LSV of bare Zn and Zn-SF electrodes (**a**) and the corresponding Tafel curves (**b**). Elution profiles of $H_2$ gases (**c**) during the stripping/plating of bare Zn and Zn-SF electrodes. Here the trace $O_2$ is probably due to the residual $O_2$ in the reactor

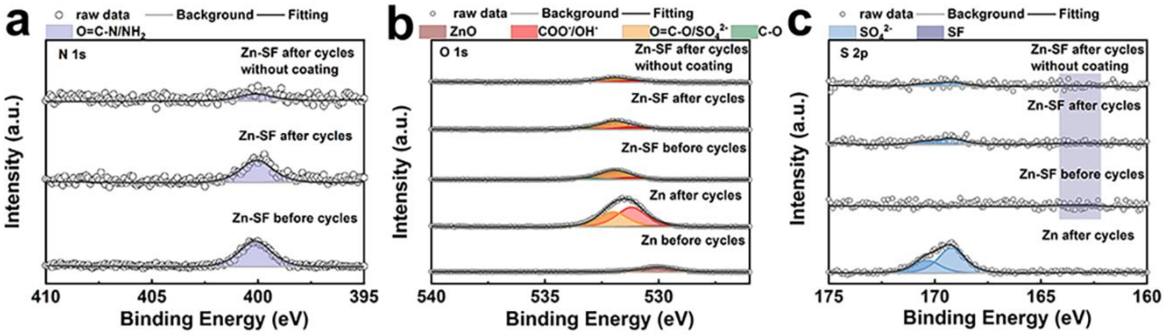


**Fig. S13** N 1s (**a**), O 1s (**b**) and S 2p (**c**) XPS spectra of two electrodes before/after cycles

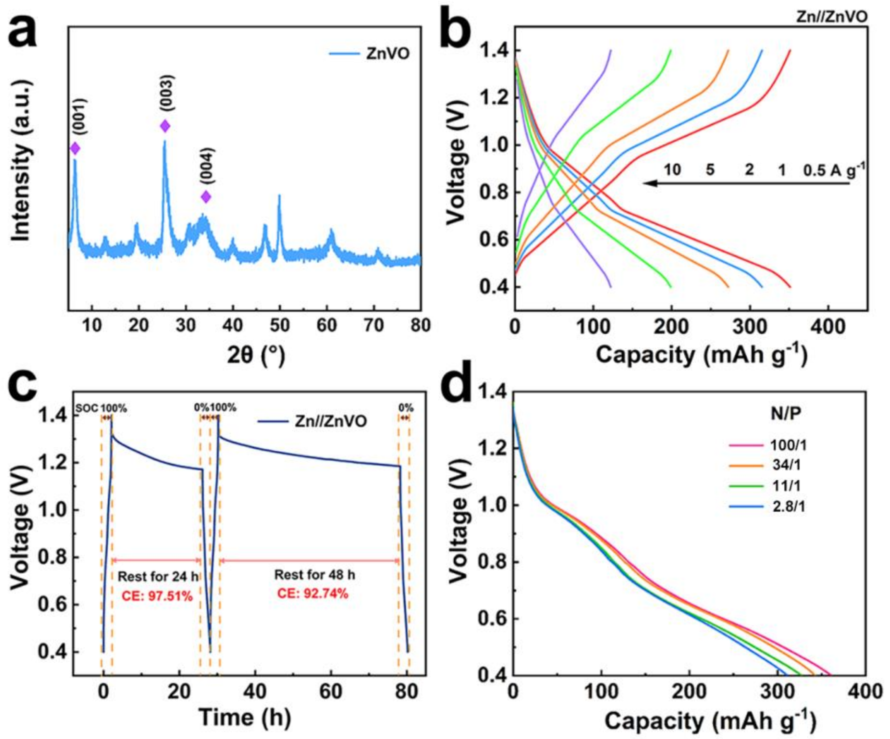


**Fig. S14** (**a**) XRD pattern of ZnVO. (**b**) Charging/discharging curves of Zn//ZnVO cell at various current densities. (**c**) Evaluation of self-discharging level for Zn//ZnVO cell rested at 100% stage of charge for 24 and 48 h. (**d**) Discharging curves of Zn-SF//ZnVO at 0.5 A $g^{-1}$ with different N/P ratios

The energy density ($E$) based on the electrode and electrode+electrolyte was calculated according to Eq. S5:

$$E = \frac{\int Q dV}{m} \tag{S5}$$

where $Q$ and $V$ represented the discharging specific capacity and voltage, respectively. $m$ was the mass of electrode/electrode+electrolyte, and $t$ was the discharging time.

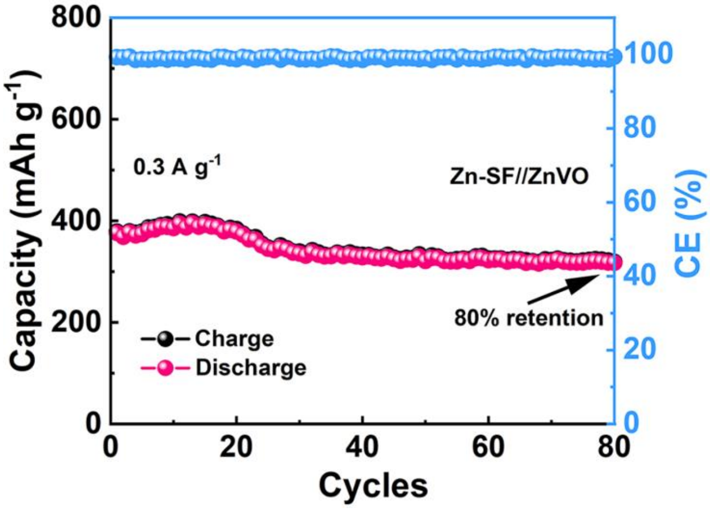


**Fig. S15** GCD cycles of Zn-SF//ZnVO full cells at 300 mA $g^{-1}$

**Table S1** Performance comparisons of the reported aqueous zinc ion batteries

| Anode//Cathode | Current density ($A\ g^{-1}$) | Specific capacity ($mAh\ g^{-1}$) | Cycles (n) | Retention (%) | Refs. |
|---|---|---|---|---|---|
| PZIL-Zn//$MnO_2$ | 0.7 | 220 | 500 | 94 | [S1] |
| Zn@PFSA//$MnO_2$ | 0.5 | 211 | 500 | 100 | [S4] |
| Zn-PA// $MnO_2$ | 0.6 | 176 | 1000 | 88 | [S5] |
| Zn-G//$V_2O_5·xH_2O$ | 5 | 120 | 1500 | 84 | [S7] |
| Zn-Gel// $V_6O_{13}$ | 0.1 | 380 | 250 | 83 | [S11] |
| Zn@$CeO_2$/$MoS_2$ | 2 | 110 | 1000 | 77 | [S15] |
| Zn@Mxene/$MnO_2$ | 1 | 253 | 500 | 81 | [S17] |
| Zn//LFP | 0.5 | 110 | 500 | 64 | [S18] |
| Zn-SF//ZnVO | 5<br>0.3 | 220<br>398 | 3000<br>80 | 80.3<br>80 | This work |

## Supplementary References


[S1] R. Chen, Q. Liu, L. Xu, X. Zuo, F. Liu et al., Zwitterionic bifunctional layer for reversible Zn anode. ACS Energy Lett. **7**(5), 1719-1727 (2022). https://doi.org/10.1021/acsenergylett.2c00124

[S2] J. Zheng, Z. Cao, F. Ming, H. Liang, Z. Qi et al., Preferred orientation of tin coatings enables stable zinc anodes. ACS Energy Lett. **7**(1), 197-203 (2021). https://doi.org/10.1021/acsenergylett.1c02299

[S3] S. Jiao, J. Fu, M. Wu, T. Hua, H. Hu, Ion sieve: tailoring $Zn^{2+}$ desolvation kinetics and flux toward dendrite-free metallic zinc anodes. ACS Nano **16**(1), 1013-1024 (2021). https://doi.org/10.1021/acsnano.1c08638

[S4] L. Hong, X. Wu, L.Y. Wang, M. Zhong, P. Zhang et al., Highly reversible zinc anode enabled by a cation-exchange coating with Zn-ion selective channels. ACS Nano **16**(4), 6906-6915 (2022). https://doi.org/10.1021/acsnano.2c02370

[S5] Z. Zhao, J. Zhao, Z. Hu, J. Li, J. Li et al., Long-life and deeply rechargeable aqueous Zn anodes enabled by a multifunctional brightener-inspired interphase. Energy Environ. Sci. **12**(6), 1938-1949 (2019). https://doi.org/10.1039/c9ee00596j

[S6] M. Liu, J. Cai, H. Ao, Z. Hou, Y. Zhu et al., $NaTi_2(PO_4)_3$ solid-state electrolyte protection layer on Zn metal anode for superior long-life aqueous zinc-ion batteries. Adv. Funct. Mater. **30**(50), 2004885 (2020). https://doi.org/10.1002/adfm.202004885

[S7] Z. Li, L. Wu, S. Dong, T. Xu, S. Li et al., Pencil drawing stable interface for reversible and durable aqueous zinc-ion batteries. Adv. Funct. Mater. **31**(4), 2006495 (2020). https://doi.org/10.1002/adfm.202006495

[S8] Y. Jiao, F. Li, X. Jin, Q. Lei, L. Li et al., Engineering polymer glue towards 90% zinc utilization for 1000 hours to make high-performance Zn-ion batteries. Adv. Funct. Mater. **31**(49), 2107652 (2021). https://doi.org/10.1002/adfm.202107652

[S9] J. Hao, X. Li, S. Zhang, F. Yang, X. Zeng et al., Designing dendrite-free zinc anodes for advanced aqueous zinc batteries. Adv. Funct. Mater. **30**(30), 2001263 (2020). https://doi.org/10.1002/adfm.202001263

[S10] L. Kang, M. Cui, F. Jiang, Y. Gao, H. Luo et al., Nanoporous $CaCO_3$ coatings enabled uniform Zn stripping/plating for long-life zinc rechargeable aqueous batteries. Adv. Energy Mater. **8**(25), 1801090 (2018). https://doi.org/10.1002/aenm.201801090

[S11] J. Shin, J. Lee, Y. Kim, Y. Park, M. Kim et al., Highly reversible, grain-directed zinc deposition in aqueous zinc ion batteries. Adv. Energy Mater. **11**(39), 2100676 (2021). https://doi.org/10.1002/aenm.202100676

[S12] M. Zhu, J. Hu, Q. Lu, H. Dong, D.D. Karnaushenko et al., A patternable and in situ formed polymeric zinc blanket for a reversible zinc anode in a skin-mountable microbattery. Adv. Mater. **33**(8), 2007497 (2021). https://doi.org/10.1002/adma.202007497

[S13] T. Chen, F. Huang, Y. Wang, Y. Yang, H. Tian et al., Unveiling the synergistic effect of ferroelectric polarization and domain configuration for reversible zinc metal anodes. Adv. Sci. **9**(14), 2105980 (2022). https://doi.org/10.1002/advs.202105980

[S14] P. Chen, X. Yuan, Y. Xia, Y. Zhang, L. Fu et al., An artificial polyacrylonitrile coating layer confining zinc dendrite growth for highly reversible aqueous zinc-based batteries. Adv. Sci. **8**(11), 2100309 (2021). https://doi.org/10.1002/advs.202100309

[S15] H. Liu, J.G. Wang, W. Hua, H. Sun, Y. Huyan et al., Building ohmic contact interfaces toward ultrastable Zn metal anodes. Adv. Sci. **8**(23), 2102612 (2021). https://doi.org/10.1002/advs.202102612

[S16] H. Yang, Z. Chang, Y. Qiao, H. Deng, X. Mu et al., Constructing a super-saturated electrolyte front surface for stable rechargeable aqueous zinc batteries. Angew. Chem. Int. Ed. **59**(24), 9377-9381 (2020). https://doi.org/10.1002/anie.202001844

[S17] N. Zhang, S. Huang, Z. Yuan, J. Zhu, Z. Zha et al., Direct self-assembly of MXene on Zn anodes for dendrite-free aqueous zinc-ion batteries. Angew. Chem. Int. Ed. **60**(6), 2861-2865 (2021). https://doi.org/10.1002/anie.202012322

[S18] J. Hao, J. Long, B. Li, X. Li, S. Zhang et al., Toward high-performance hybrid Zn-based batteries via deeply understanding their mechanism and using electrolyte additive. Adv. Funct. Mater. **29**(34), 1903605 (2019). https://doi.org/10.1002/adfm.201903605
